# Supplementary material for: Curvature-based sorting of eight lipid types in asymmetric buckled plasma membrane models
Source: Biophys J. 2022 May 5;121(11):2060–8. doi: 10.1016/j.bpj.2022.05.002 (PMC9247473; doi:10.1016/j.bpj.2022.05.002)
Supplement: Document S1. Figures S1–S3 and Tables S1–S6 [file mmc1.pdf]

Biophysical Journal, Volume *121*

Supplemental information

Curvature-based sorting of eight lipid types in asymmetric buckled plasma membrane models

Elio A. Cino and D. Peter Tieleman

Curvature-based sorting of eight lipid types in asymmetric buckled plasma membrane models

Elio A. Cino[1], D. Peter Tieleman[1*]

[1]Centre for Molecular Simulation and Department of Biological Sciences, University of Calgary, 2500 University Drive NW, Calgary, Alberta T2N 1N4, Canada

***D. Peter Tieleman, tieleman@ucalgary.ca**

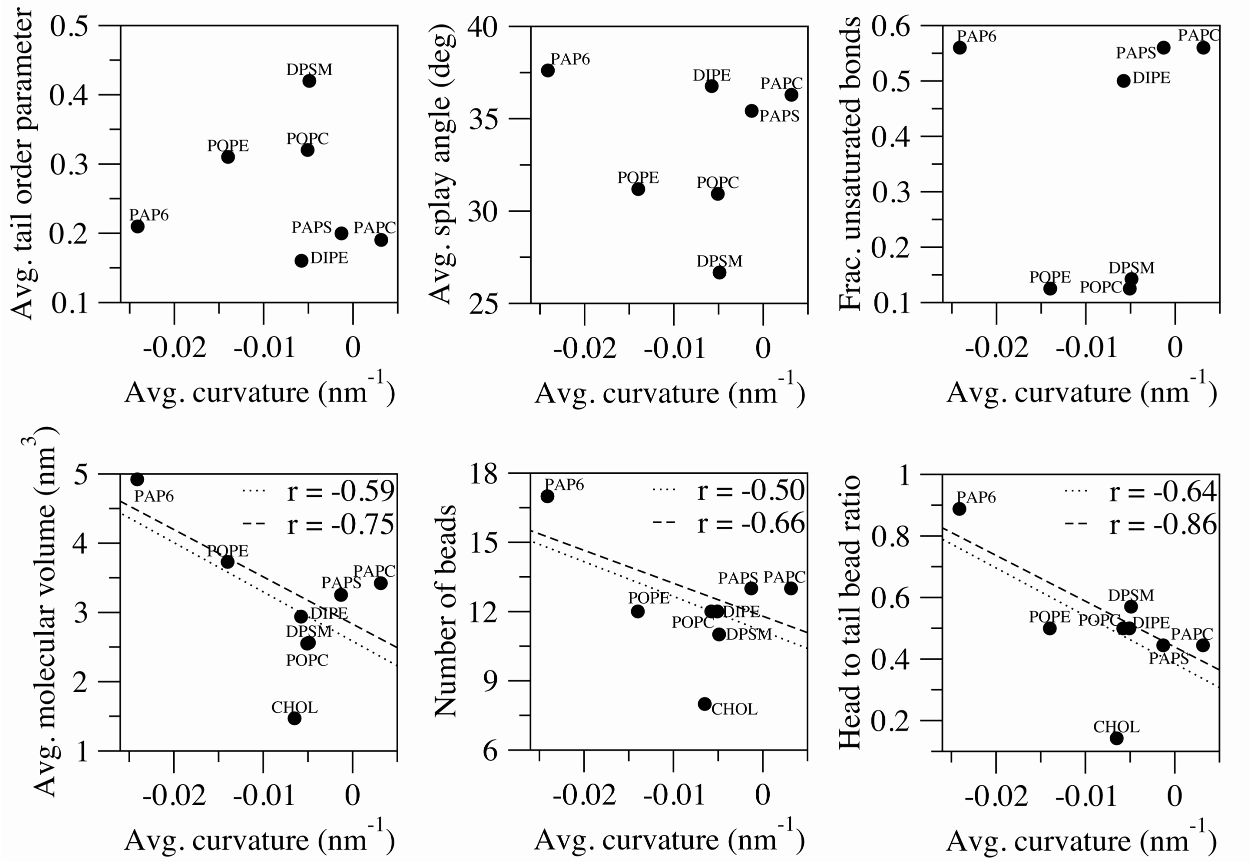

Figure S1. Correlations between average curvatures and various parameters. Splay angles were defined by the terminal beads of each tail and PO4 of the headgroup, similar to (1). Absolute lipid tail order parameters (average of the tail beads) were calculated using the do-order-gmx5.py script from the Martini website. Approximate lipid molecular volumes were computed using gmx sasa with a probe radius of 0.26 nm, which corresponds to the bead vdW radius (2). The values were systematically higher than reported from experiment (3); however the relative values are consistent with the molecular structures (Fig. 1), and similar trends were obtained using the number of beads instead of volumes. Dotted and dashed lines correspond to fits including and excluding CHOL, respectively.

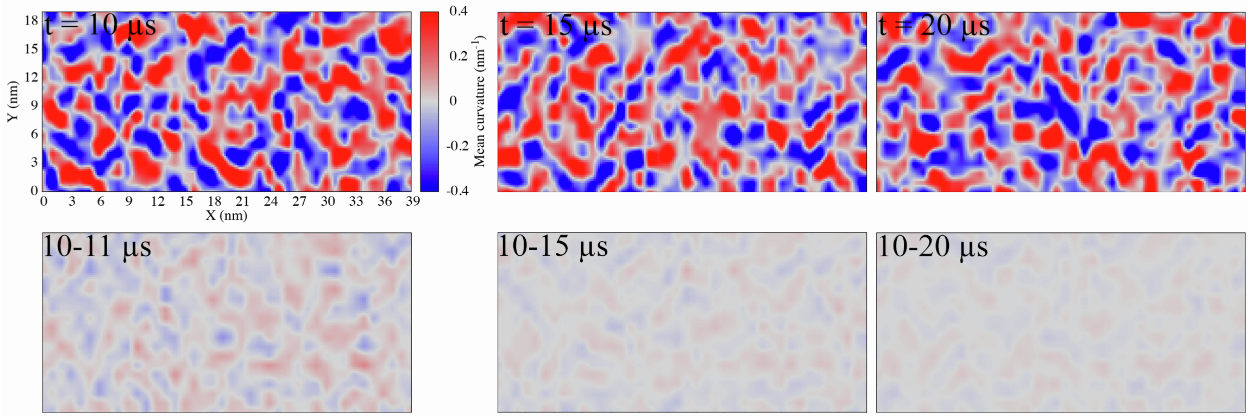

Figure S2. Mean curvature maps of the outer leaf of the unbuckled system. Single frame calculations at 10, 15 and 20 μs (top), and averaging over 1, 5, and 10 μs time intervals (bottom).

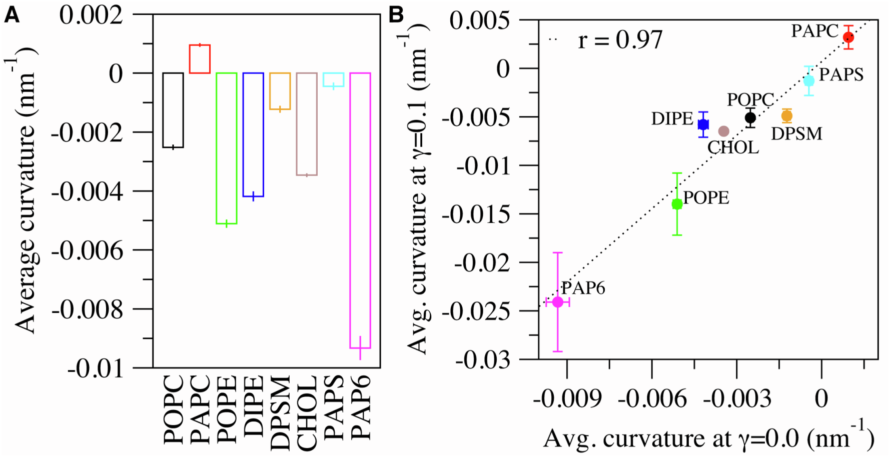

Figure S3. Curvature values of lipids in the unbuckled system and comparison with a buckled system. A) Mean curvature values of lipids in the unbuckled, γ=0.0 system. B) Comparison of the curvature values from the γ=0.0 and γ=0.1 systems.

Table S1: Average number of lipid-lipid contacts in the γ=0.0 outer leaf.

	POPC	PAPC	POPE	DIPE	DPSM
POPC	0.85±0.01	0.65±0.01	0.26±0.01	0.53±0.01	0.86±0.01
PAPC		0.83±0.01	0.37±0.02	0.87±0.02	0.60±0.01
POPE			1.02±0.03	0.64±0.02	0.27±0.01
DIPE				1.33±0.04	0.46±0.01
DPSM					0.89±0.01

Table S2: Average number of lipid-lipid contacts in the γ=0.0 inner leaf.

	POPC	PAPC	POPE	DIPE	DPSM	PAPS	PAP6
POPC	0.98±0.01	0.70±0.01	0.88±0.01	0.79±0.01	0.98±0.01	0.84±0.01	0.38±0.01
PAPC		0.83±0.01	0.79±0.02	0.77±0.01	0.72±0.01	0.74±0.01	0.52±0.02
POPE			1.18±0.03	0.62±0.01	0.96±0.02	0.67±0.01	1.01±0.05
DIPE				1.14±0.01	0.68±0.01	0.94±0.01	0.54±0.01
DPSM					1.04±0.02	0.78±0.01	0.43±0.02
PAPS						0.70±0.01	0.22±0.01
PAP6							1.53±0.09

Table S3: Average number of lipid-lipid contacts in the γ=0.1 outer leaf.

	POPC	PAPC	POPE	DIPE	DPSM
POPC	0.87±0.01	0.65±0.01	0.28±0.01	0.53±0.01	0.86±0.01
PAPC		0.83±0.02	0.37±0.02	0.88±0.02	0.60±0.01
POPE			1.09±0.24	0.65±0.03	0.27±0.01
DIPE				1.36±0.08	0.46±0.01
DPSM					0.89±0.01

Table S4: Average number of lipid-lipid contacts in the γ=0.1 inner leaf.

	POPC	PAPC	POPE	DIPE	DPSM	PAPS	PAP6
POPC	0.99±0.01	0.70±0.01	0.88±0.01	0.79±0.01	1.00±0.01	0.85±0.01	0.39±0.02
PAPC		0.84±0.02	0.79±0.01	0.77±0.01	0.71±0.01	0.75±0.01	0.51±0.01
POPE			1.19±0.03	0.64±0.01	0.97±0.03	0.67±0.01	1.07±0.06
DIPE				1.16±0.01	0.68±0.01	0.94±0.01	0.57±0.03
DPSM					1.06±0.01	0.78±0.01	0.44±0.03
PAPS						0.70±0.01	0.22±0.01
PAP6							1.51±0.14

Table S5: Average number of lipid-lipid contacts in the γ=0.4 outer leaf.

	POPC	PAPC	POPE	DIPE	DPSM
POPC	0.90±0.01	0.64±0.01	0.30±0.01	0.56±0.01	0.89±0.01
PAPC		0.81±0.02	0.35±0.01	0.85±0.02	0.60±0.01
POPE			1.21±0.18	0.74±0.05	0.28±0.01
DIPE				1.42±0.04	0.48±0.01
DPSM					0.90±0.01

Table S6: Average number of lipid-lipid contacts in the γ=0.4 inner leaf.

	POPC	PAPC	POPE	DIPE	DPSM	PAPS	PAP6
POPC	0.99±0.01	0.69±0.01	0.90±0.01	0.82±0.01	0.99±0.01	0.84±0.01	0.40±0.01
PAPC		0.81±0.01	0.77±0.02	0.74±0.01	0.72±0.01	0.73±0.01	0.48±0.03
POPE			1.27±0.08	0.69±0.02	0.97±0.01	0.65±0.01	1.29±0.17
DIPE				1.25±0.04	0.68±0.01	0.93±0.01	0.71±0.04
DPSM					1.04±0.02	0.77±0.01	0.42±0.03
PAPS						0.70±0.01	0.23±0.01
PAP6							2.01±0.32

References

1. Kawamoto, S., M.L. Klein, and W. Shinoda. 2015. Coarse-grained molecular dynamics study of membrane fusion: Curvature effects on free energy barriers along the stalk mechanism. *J. Chem. Phys.* 143:243112.
2. Periole, X., A.M. Knepp, T.P. Sakmar, S.J. Marrink, and T. Huber. 2012. Structural determinants of the supramolecular organization of G protein-coupled receptors in bilayers. *J. Am. Chem. Soc.* 134:10959–10965.
3. Marsh, D. 2010. Molecular volumes of phospholipids and glycolipids in membranes. *Chem. Phys. Lipids*. 163:667–677.
