# Supplementary material for: Curvature-based sorting of eight lipid types in asymmetric buckled plasma membrane models
Source: Biophys J. 2022 May 5;121(11):2060–8. doi: 10.1016/j.bpj.2022.05.002 (PMC9247473; doi:10.1016/j.bpj.2022.05.002)
Supplement: Document S2. Article plus supporting material [file mmc2.pdf]

Biophysical Journal
Article

Biophysical Society

Curvature-based sorting of eight lipid types in asymmetric buckled plasma membrane models

Elio A. Cino[1] and D. Peter Tieleman[1,*]
[1]Centre for Molecular Simulation and Department of Biological Sciences, University of Calgary, Calgary, Canada

ABSTRACT Curvature is a fundamental property of biological membranes and has essential roles in cellular function. Bending of membranes can be induced by their lipid and protein compositions, as well as peripheral proteins, such as those that make up the cytoskeleton. An important aspect of membrane function is the grouping of lipid species into microdomains, or rafts, which serve as platforms for specific biochemical processes. The fluid mosaic model of membranes has evolved to recognize the importance of curvature and leaflet asymmetry, and there are efforts toward evaluating their functional roles. This work investigates the effect of curvature on the sorting of lipids in buckled asymmetric bilayers containing eight lipid types, approximating an average mammalian plasma membrane, through coarse-grained (CG) molecular dynamics (MD) simulations with the Martini force field. The simulations reveal that 1) leaflet compositional asymmetry can induce curvature asymmetry, 2) lipids are sorted by curvature to different extents, and 3) curvature-based partitioning trends show moderate to strong correlations with lipid molecular volumes and head to tail bead ratios, respectively. The findings provide unique insights into the role of curvature in membrane organization, and the curvature-based sorting trends should be useful references for later investigations and potentially interpreting the functional roles of specific lipids.

SIGNIFICANCE Membrane curvature is a fundamental aspect of living organisms that is central to numerous biochemical processes. Bending of membranes has long been observed, but the molecular details of how different lipids organize and function in curved membranes are not fully understood. Molecular dynamics simulations have proven to be a crucial tool for obtaining such information. Our simulations show that leaflet compositional asymmetry can induce curvature asymmetry, that different lipids can have unique curvature preferences, and that the curvature partitioning trends correlate with head to tail size ratios. The findings provide insights into the role of curvature in membrane organization and potentially explain the functional roles of specific lipids.

INTRODUCTION

Biological membranes fulfill numerous functions and are crucial for the viability of cells. In addition to providing a selective barrier to the extracellular environment, they permit separation of distinct functions carried out by membrane-bound organelles within eukaryotic cells (1). The lipid composition of biomembranes can be highly diverse, giving rise to specific physical properties and functionalities (2). One such property is curvature. Although the bending of membranes has long been observed, and its general importance recognized, the molecular details of how different lipids and proteins distribute and function in curved membranes are not well understood (3).

Submitted February 8, 2022, and accepted for publication May 2, 2022.
*Correspondence: tieleman@ucalgary.ca
Editor: Heiko Heerklotz.
https://doi.org/10.1016/j.bpj.2022.05.002
© 2022 Biophysical Society.
This is an open access article under the CC BY license (http://creativecommons.org/licenses/by/4.0/).

Membrane curvature is a central aspect of virtually all cells. Processes fundamental to life, such as cytokinesis, membrane remodeling, and vesicle formation involve curvature (4). In eukaryotes, organelles display a large variability in the curvature of their membranes, which is key to their distinct functions. For instance, the highly folded structure of the inner mitochondrial membrane is crucial for increasing the surface area available for proteins involved in aerobic respiration (5). Likewise, tight bending of thylakoid membranes is required for the formation of grana stacks in photosynthetic organisms (6).

Due to the intimate relationship between membrane curvature and biological function, it is important to understand how it is generated, regulated, and its context-dependant purpose. As reviewed in detail by McMahon and Boucrot (7), membrane curvature is prevalent in cells and arises in numerous ways, including the lipid and protein composition of membranes, peripheral proteins, and those that make up the

cytoskeleton. Cylindrically shaped lipids such as phosphatidylcholine and phosphatidylserine tend to form flat membranes, whereas those with disproportionate head to tail dimensions, such as phosphatidylethanolamine and phosphatidylinositol phosphates, may promote membrane curvature or preferentially localize to curved regions. Other factors, like the extent of tail unsaturation and lipid-lipid and lipid-protein interactions also play a role in regulating lipid localization. By controlling lipid composition, for instance through the regulation of uptake and synthesis, cells and individual organelles can modulate the curvature of their membranes. Integral membrane proteins can also shape membranes (8), as can membrane-binding proteins, such as BAR domains, which impose local curvature through interactions with charged lipid headgroups (9). Cytoskeletal proteins that make up filopodia and lamellipodia can dynamically control vesicle formation, membrane protrusions, such as those involved in cell migration, and create distinct cell morphologies like neutrophils and neurons (10).

Though the ability of cells to generate and regulate curvature is plainly evident, and many of the mechanisms for doing so are established, molecular details on the relationship between membrane curvature and function are less explored. Curvature is often thought of as part of the lipid-raft hypothesis (11,12). Lipid rafts are local microdomains in the bilayer consisting of certain lipid and protein entities that fulfill a particular function, such as a specific signaling pathway (13). Demonstration that membrane curvature alone, in the absence of proteins, is sufficient to trigger lipid sorting (14), supports a possible role of curvature in lipid-raft assembly. Moreover, it has been shown that the intrinsic shape of lipids is sufficient to promote clustering at specific regions of curvature so long as the enthalpic gains of demixing are greater than the entropic loss (15). Protein sorting as a function of curvature has also been reported. A prominent example is the clustering of mitochondrial ATP synthase dimers along the highly curved crista ridges or tubular cristae and depletion in the flat stretches of inner membrane between cristae (16,17). Molecular-dynamics (MD) simulations have shown that ATP synthase dimers can induce curvature of flat POPC bilayers, supporting the proposal that they are crucial for cristae morphogenesis (18). Still, many aspects of ATP synthase function in relation to curvature remain under investigation, like how the dimers remain anchored at specific membrane regions and whether or not the curved environment influences its catalytic activity (19). Curvature-driven modulation of protein function has been demonstrated in a few cases. The specific activity of diacylglycerol kinase ε can be over 10-fold higher in negatively curved versus flat membranes without preferential binding to one form over the other (20). Furthermore, conformational changes during photoactivation of rhodopsin become more favorable as the curvature elastic energy of the membrane increases (21). Estimates of membrane curvature free energy are well within the range required for protein conformational changes, suggesting that the elastic energy stored in the membrane may be able to allosterically regulate a vast assortment of membrane proteins (22–24).

This work aims to assess the effects of membrane curvature on the distributions of lipids in an asymmetric plasma membrane model with eight lipid types (Fig. 1), including cholesterol, with a composition approximating that of an average mammalian cell. Considerable variation in curvature partitioning was observed between the different lipid types, and the curvature-based sorting trends showed weak to strong correlations with different physical parameters of the lipids. While good consistency with reported curvature-partitioning tendencies of specific lipids was seen, it is likely that the sorting behaviors have some level of system dependence, necessitating reassessment for different membrane compositions.

MATERIALS AND METHODS

System preparation

Solvated 40*20*20 nm asymmetric bilayer systems containing eight lipid types (Table 1), corresponding to an average plasma membrane composition (25), were built using insane (26). Plasma membranes typically contain hundreds of different lipid types; however, reproducing this level of complexity in simulations is difficult due to numerous factors, such as

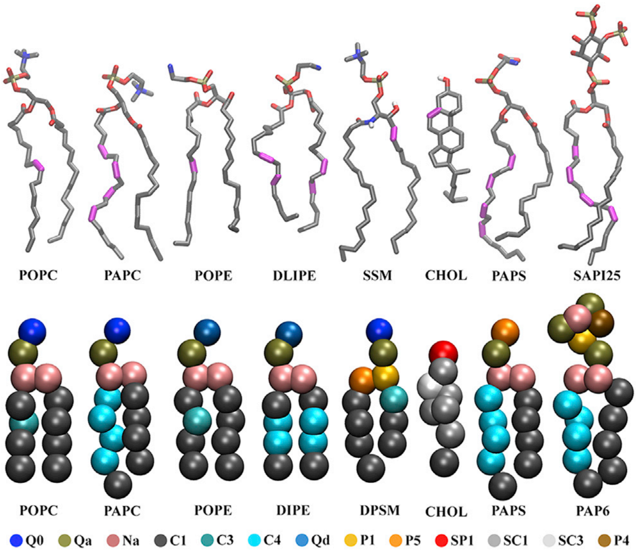

FIGURE 1 Atomistic and coarse-grained (CG) representations of the lipid species in this study. (*Top*) Atomistic lipid structures with their CHARMM36 residue names, and unsaturated bonds in the tails colored magenta. (*Bottom*) CG Martini 2.2 models of the lipids colored by bead type. Abbreviation key for the CG lipid names: POPC, 1-palmitoyl-2-oleoyl-sn-glycero-3-phosphocholine; PAPC, 1-palmitoyl-2-arachidonyl-sn-glycero-3-phosphatidylcholine; POPE, 1-palmitoyl-2-oleoyl-sn-glycero-3-phosphoethanolamine; DIPE, 1,2-dilinoleoyl-sn-glycero-3-phosphoethanolamine; DPSM, N-stearoyl-D-erythro-sphingosylphosphorylcholine; CHOL, cholesterol; PAPS, 1-palmitoyl-2-arachidonyl-phosphatidylserine; PAP6, phosphatidylinositol 4,5-bisphosphate. To see this figure in color, go online.

TABLE 1 Membrane leaflet lipid fractional compositions and (counts)[a]

	POPC	PAPC	POPE	DIPE	DPSM	PAPS	PAP6[b]	CHOL
Outer	0.243 (328)	0.121 (163)	0.020 (27)	0.061 (82)	0.242 (327)	0.000 (0)	0.000 (0)	0.313 (423)
Inner	0.139 (187)	0.075 (101)	0.054 (73)	0.161 (217)	0.108 (146)	0.161 (217)	0.022 (29)	0.280 (378)

[a]The outer and inner leaflets contain 1350 and 1348 total lipids, respectively.
[b]Parameters for PAP6, a PIP2 lipid, were generated using the Martini lipid itp generator script from the Martini website with -alhead 'P2' -allink 'G G' -altail 'CCCC DDDDC' (25,26).

the availability of parameters and balance of system size with adequate sampling and computational time. The eight-lipid-type model used here was iteratively developed to recapture the lipid headgroup and tail distributions of a "gold standard" 60-lipid-species model (27,28). The compositional fractions used give a good balance in lipid packing between leaflets, well within a tolerable degree of mismatch (29). It is important to avoid excessive differences in the number of lipids per leaflet in bilayer simulations to minimize packing defects, which can result in spontaneous curvature due to differential stress (30). All species were represented using the Martini 2.2 parameter set (31). To prevent the freezing of water, 10% of the solvent was antifreeze particles (32). Charge neutrality and a physiological ion concentration of 0.15 M was achieved using Na^+ and Cl^- particles. A bilayer with a symmetric lipid distribution (both leaflets with the inner leaf composition; Table 1) was included as a control.

MD simulations

Energy minimization and stepwise equilibration of the systems was carried out with GROMACS 2019 (33) using the "martini_v2.x_new-rf.mdp" run parameters (34) with a 20-fs timestep. Temperatures of the lipid and solvent (water, antifreeze, and ion) beads were controlled separately at 310 K using the velocity-rescaling thermostat (35) with a time constant of 1 ps. The Parrinello-Rahman algorithm (36) was used for semi-isotropic pressure coupling at 1 bar, with a time constant of 12 ps and a compressibility of 3×10^{-4} bar^{-1}. The Verlet cutoff scheme was used, and van der Waals (cutoff) and Coulomb (reaction-field with electrostatic screening constant of 15) interactions were set to zero at 1.1 nm. The equilibrated systems were propagated at 310 K for 2 μs at 1 bar pressure to establish equilibrium lipid distributions (37). Next, a gradual pressure increment up to 4 bar was applied along the long (X) dimension to generate buckled membranes with different extents of compressional strain, γ:

$$\gamma = \frac{X_0 - X_i}{X_0}$$

where X_0 and X_i are the box lengths along the X dimension before and after buckling, respectively. System coordinates at $\gamma = 0.0$, 0.1, 0.2, 0.3, and 0.4 (Fig. 2 *A*) were extracted and used to initiate NVT simulations at constant compressional strain, which were propagated at 310 K for 20 μs at each compression level.

Data analysis

The pointwise curvature $C(x)$ of each leaflet was calculated by fitting a Bezier spline on the lipid headgroup positions in the XZ plane (Fig. 2 *B*) and computing the first and second derivatives:

$$C(x) = \frac{f''(x)}{\left(1 + f'(x)^2\right)^{\frac{3}{2}}}$$

where C is curvature in nm^{-1} and f' and f'' are the first and second derivative of headgroup height with respect to X. The signs of curvature values for the outer leaflet were inverted, resulting in concave and convex regions being signed negative and positive, respectively (Fig. 2 *B*). Lipid fractions with respect to curvature were determined by their headgroup locations (Fig. 2 *C*), using a bin width of 0.05 nm. The 10-20 μs interval was used for analysis, and the block average of 4×2.5 μs intervals $\pm$ SD is reported.

Bending modulus calculations were performed on the equilibrated unbuckled, $\gamma = 0.0$, system trajectories using the lipid-splay method and Python implementation (38,39). All lipid species were considered in the calculation, and the overall average is reported. For the unbuckled system, mean curvature was calculated using g_lomepro (40), which utilizes a modified version of GridMAT-MD (41) to map selected lipid atoms onto a grid, and we subsequently calculated first and second order derivates over the surface for estimation of the mean curvature.

RESULTS AND DISCUSSION

Asymmetric bilayer composition led to asymmetric curvature between leaflets

Histograms of the curvature values of all lipids revealed that in the asymmetric bilayer, the inner leaf had a greater fraction of extreme curvatures than the outer leaf (Fig. 3). In contrast, the control symmetric bilayer had nearly equal curvature distributions between leaflets. Boyd et al. also observed more extreme curvatures in the inner versus outer leaf in an asymmetric bilayer composed of 50% POPC and 50% POPE in the outer leaf and 40% POPC, 40% POPE, and 20% cardiolipin in the inner leaf (42). In the aforementioned study, cardiolipin, a tetra-acyl lipid, was found to preferentially accumulate in regions of high negative curvature. The bilayer composition used in this work contained only diacyl phospholipids and cholesterol, suggesting that other lipid types and asymmetric bilayer compositions may lead to a similar phenomenon, which is discussed further in the following sections. Compositional asymmetry is crucial for numerous aspects of cell homeostasis, such as generation of charge gradients, targeting, and signal transduction. Asymmetric leaflet composition can also induce membrane curvature (43). A number of studies have pointed to a link between curvature asymmetry and regulation of membrane mechanics, which affects many biochemical processes such as localization and activity of integral membrane proteins (44). For example, spontaneous curvature generation when the GM1 glycolipid is distributed asymmetrically across a model membrane is thought to be important for shaping of neuronal morphology (45). Moreover, experiments with giant unilamellar vesicles (GUVs) suggest that asymmetry between leaflets can alter membrane mechanical properties, with asymmetric GUVs having a greater bending rigidity compared with symmetric GUVs (46,47). In line with these demonstrations, the bending modulus of the unbuckled asymmetric and symmetric bilayers studied here were 25.0 $\pm$ 0.2 and 21.2 $\pm$ 0.1 k_BT,

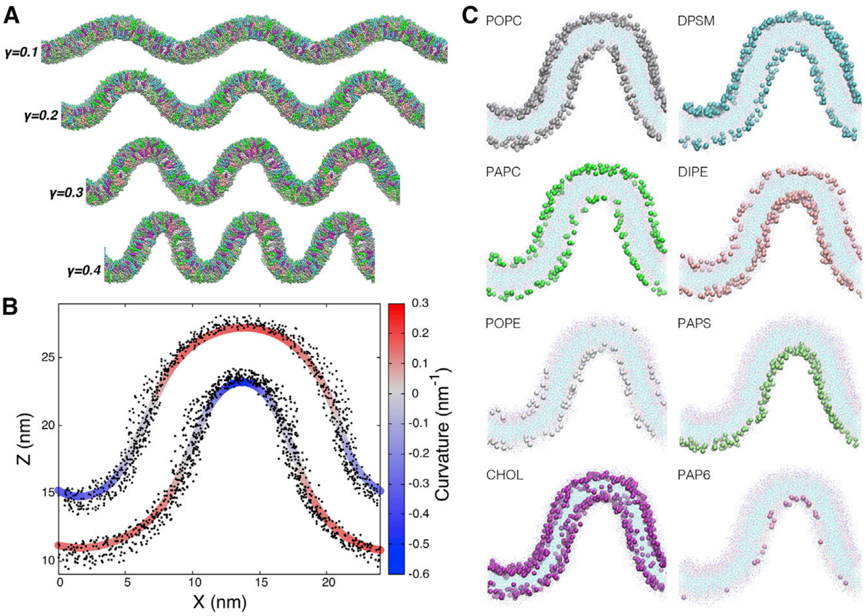

FIGURE 2 Overview of the systems and curvature-partitioning analysis. (*A*) Illustration of the different compression levels. (*B*) Spline fits through the lipid headgroup positions colored by curvature for the $\gamma = 0.4$ system. Note that the observed curvature is dependent on the head group bead used. (*C*) Distributions of the different lipid types at $\gamma = 0.4$ after 10 μs. To see this figure in color, go online.

respectively, which are within the expected range for mammalian cell membranes (48). While the difference in bending modulus between asymmetric and symmetric bilayers may not seem large, it could still be relevant. Similar variations in bending modulus have been shown to correspond to changes in cell geometry, lipid domain structure, membrane thickness, and the activity of membrane proteins (48–50). Moreover, considering that cells actively regulate and maintain compositional asymmetry between leaflets, it seems plausible that, in addition to previously ascribed functions, increasing membrane elastic energy may also be favored.

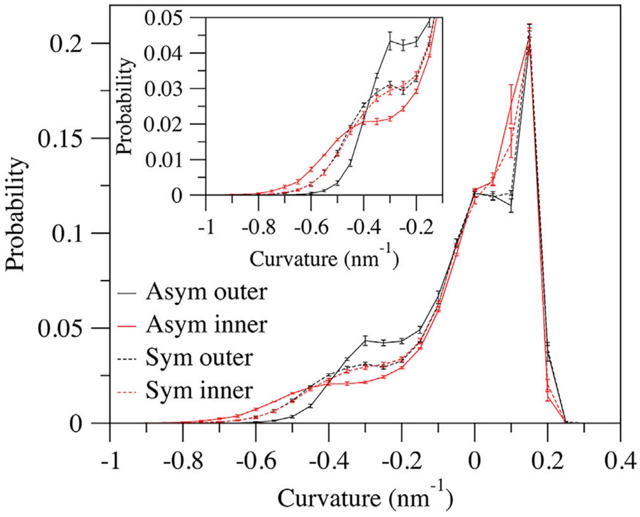

FIGURE 3 Lipid curvature distributions in asymmetric and symmetric bilayers. The inset shows a focused view of the -0.3 to -1 nm^{-1} region. Curvature values of all lipids from the $\gamma = 0.4$ systems were considered in the analysis. The block average of 4×2.5 μs intervals ± SD is reported. To see this figure in color, go online.

Lipids sorted by curvature to different extents

As shown in Fig. 4, the lipids showed varying degrees of curvature partitioning, with highly similar trends across different levels of buckling. Given the consistency of the curvature partitioning trends, subsequent analysis focused on the 0.1 compression level, which gives rise to a high proportion of curvature values in the most biologically relevant range of 0.02–0.13 nm^{-1} (51). Linear regression analysis of the distributions in Fig. 4 was used to assess the extent of curvature partitioning for each lipid type (Table 2). PAP6, a PIP2 lipid, and cholesterol showed the highest and lowest extents of curvature partitioning, respectively (Fig. 4; Table 2).

The average curvature values (Fig. 5 *A*, *top panel*) show that PAP6 sorted to regions of negative curvature to the greatest extent of all the lipids, followed by POPE. Consistent with our findings, other studies have also reported PIP2 and POPE enrichment in curved regions and relatively curvature agnostic behavior for POPC, PS, and SM lipids (52). Fig. 5 *B* illustrates where the lipids tended to localize across the buckled membrane and provides additional insights into the partitioning trends. For instance, it has been found that phosphatidylethanolamine lipids are outcompeted for regions of greatest curvature by cardiolipin (53). Similar competition may occur between POPE and PAP6 in the inner leaf. As shown in Fig. 5 *B*, POPE was enriched in the tight bend of the outer leaf but to a lesser extent in the inner leaf, possibly due to competition for the same region with PAP6, which has the greatest preference for negative curvature. PAPC also showed evidence of curvature partitioning (Fig. 4; Table 2), seemingly with a preference for regions of moderate positive curvature. Experimental data indicate that cholesterol has a

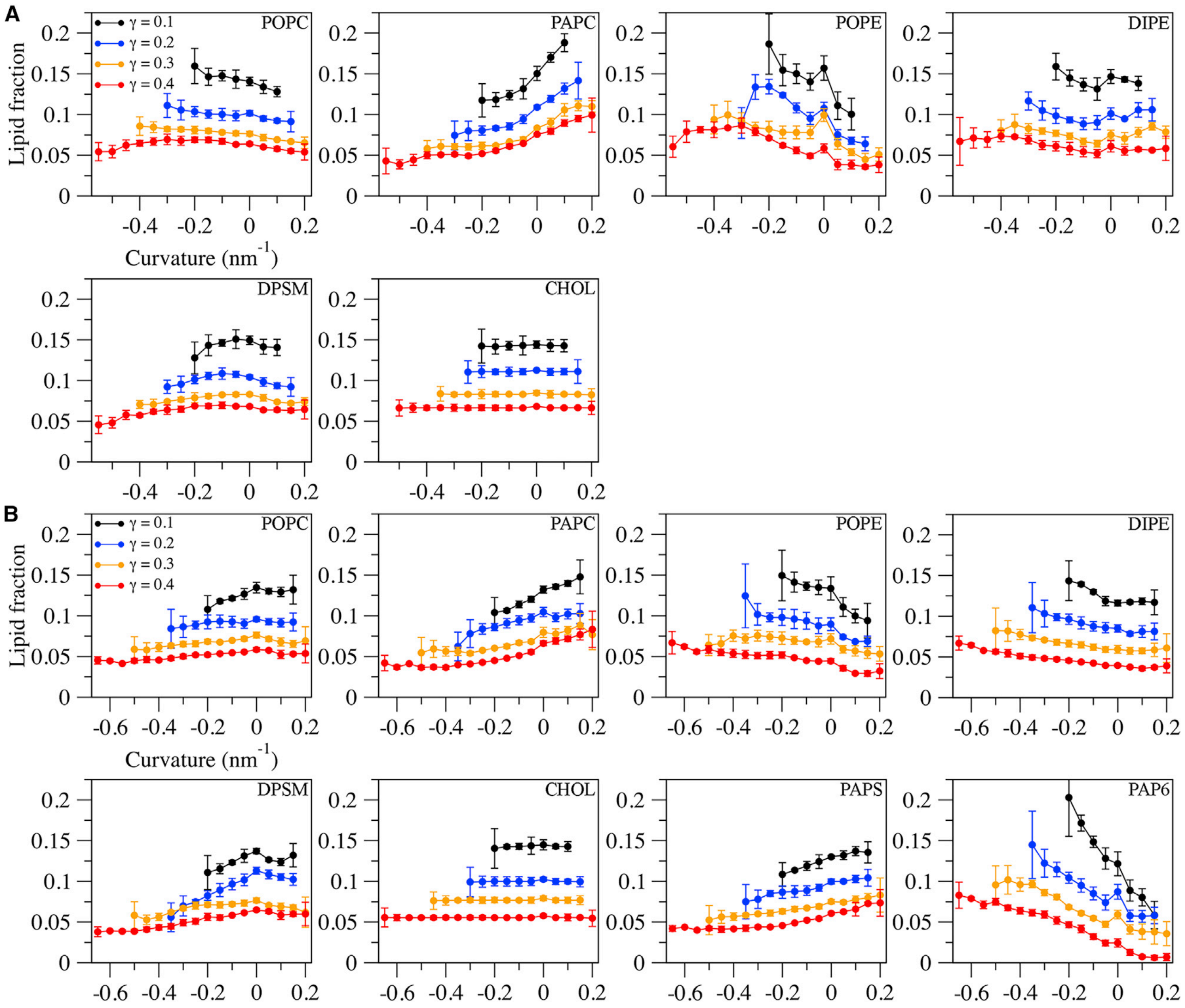

FIGURE 4 Lipid fractions as a function of curvature. (*A*) Outer leaflet lipids. (*B*) Inner leaflet lipids. Distributions were normalized by the fraction of the total number of lipids in each bin, and bins with low occupancy (<5% of total leaflet lipids) are not shown for clarity. The block average of 4 × 2.5 μs intervals ± SD is reported. To see this figure in color, go online.

preference for negative curvature (54), though in the current work and that of Baoukina et al. (55), cholesterol (CHOL) exhibited a low degree of curvature-induced sorting. The present and aforenamed research both employed Martini simulations with a similar bilayer composition, which suggests that the differences between simulation and experiment could be attributed to inherent challenges in coarse-graining molecules such as CHOL (56) or the presence of other lipid species with a stronger curvature preference. A variety of computational and experimental methods have been used to study curvature-based lipid sorting, including buckling, tethers, and pipette aspiration (42,51,55). While the particular membrane composition used here is unique from other investigations on lipid curvature partitioning, the curvature preferences that can be compared are in reasonably good agreement, and we are confident that the results are not dependent on buckling protocol used to generate curvature and should be reproducible by other approaches.

TABLE 2 Slope of the linear fit of the $\gamma = 0.1$ curvature distributions[a]

Lipid	Outer leaf	Inner leaf
POPC	-0.090 ± 0.012	0.062 ± 0.015
PAPC	0.245 ± 0.034	0.131 ± 0.007
POPE	-0.242 ± 0.054	-0.160 ± 0.020
DIPE	-0.039 ± 0.032	-0.079 ± 0.018
DPSM	0.027 ± 0.029	0.049 ± 0.021
CHOL	0.004 ± 0.003	0.007 ± 0.005
PAPS	–	0.084 ± 0.008
PAP6	–	−0.393 ± 0.021

[a]Linear regression coefficient for the $\gamma = 0.1$ datapoints from Fig. 4. Extent of deviation from zero reflects the magnitude of curvature partitioning.

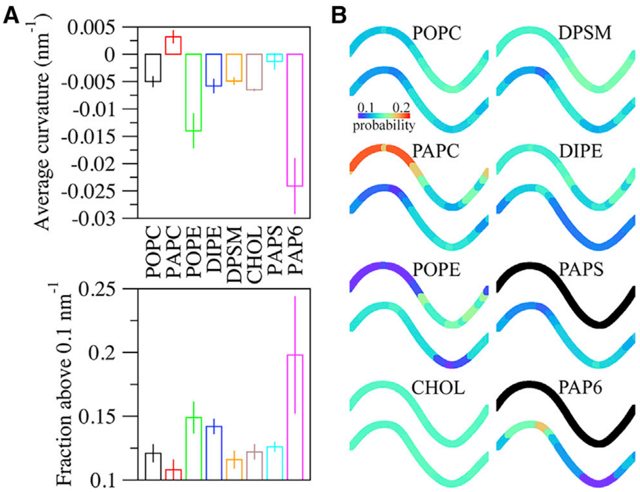

FIGURE 5 Average curvatures and lipid localization at the 0.1 compression level. (*A*) Average of the curvature values of each lipid (*top*), and fraction of absolute curvature values > 0.1 nm^{-1} (*bottom*). The block average of 4×2.5 μs intervals $\pm$ SD is reported. (*B*) Probability of lipid localization along a buckled membrane. Black shading reflects the absence of PAPS and PAP6 in the outer leaf. To see this figure in color, go online.

Assessment of the curvature-partitioning trends

To investigate the causes of the different curvature preferences, correlations between average curvature (Fig. 5 A, *top panel*) and several variables were assessed. Tail order parameter, splay angle, and extent of unsaturation correlated poorly with the curvature-partitioning trends (Fig. S1). Qualitative agreement was observed between the fraction of unsaturated tail bonds, order parameters, and splay angles. Lipids with the most unsaturated tails, including PAPC, DIPE, PAPS, and PAP6, had the lowest tail order-parameter values (least ordered tails), and were also the most splayed. DPSM had the most ordered tails and the lowest extent of splaying. Lipid molecular volumes (or simply the number of beads per lipid) had moderate correlation coefficients with the average curvature values, r = -0.75 (r = -0.66) with CHOL omitted (Fig. S2). The curvature-partitioning trends were best described by the head to tail bead ratios, r = -0.86 with CHOL excluded (Fig. S1).

In addition to the physical properties of lipids, curvature-based sorting depends on several other factors, including mixing entropy, lipid-lipid/protein interactions, and bending energy (51,57). To investigate the contribution of lipid-lipid interactions to the observed curvature-sorting trends, interaction matrices were constructed (Fig. 6). While not the primary focus here, even in the absence of buckling, the interaction profiles illustrate considerable inhomogeneity in lipid organization and evidence of domain formation, which is a key aspect of the raft hypothesis (58). The lipid-lipid interaction profiles were similar between unbuckled and buckled membranes, but some of the trends were moderately amplified with increasing curvature. PAP6 was the most prone to self interaction, followed by DIPE and POPE. Clustering of PIP2 lipids is well documented and plays an important role in numerous processes such as signaling, membrane targeting, and cytoskeletal attachment (59,60). Association of PI (phosphatidylinositol) and PE lipids has been demonstrated in small-angle X-ray scattering experiments and attributed to favorable headgroup interactions and geometric compatibility; PI/PE assemblies could serve as platforms for membrane budding and fusion (61). Elevated numbers of PAP6-POPE and DPSM-POPC contacts were also noted, while PAP6-PAPS interactions were the least frequent. It is unclear whether the aforementioned lipid-lipid contacts, or lack thereof, arose due to attractive or repulsive interactions, curvature preferences and competition, or combination of both. There were some differences in interaction patterns between the outer and inner leaflets, namely for POPE. In the outer leaf, POPE-POPC and POPE-DPSM interactions were below average but were considerably more frequent in the inner leaf. A plausible explanation is that POPE molecules tend to self associate in high curvature regions in the outer leaf but are outcompeted by PAP6 for such regions in the inner leaf (Fig. 5), leading to increased interactions with other lipids. Another factor that likely plays a role in the observed lipid-lipid interaction patterns at different curvatures and between leaflets is interleaflet coupling, where the lateral organization of lipids in one leaf influences lipid ordering in the opposing leaf. Interleaflet coupling is thought to be important for biochemical processes that require communication between the extracellular and intracellular environments and has been observed both experimentally and through simulations (62). For instance, biophysical measurements of asymmetric large unilamellar vesicles suggest that the a strong curvature preference of POPE can lead to curvature-driven interleaflet coupling (63). Together, the findings presented thus far suggest that a number of factors contribute to the curvature-partitioning trends observed here, and it is conceivable that systems with different lipid types, combinations, and ratios could show different behavior.

Finally, an evaluation of whether the lipid curvature-partitioning trends seen in buckled membranes were evident in the unbuckled state was performed. Mean curvature maps of the unbuckled system show that it exhibited spontaneous thermal fluctuations leading to transiently curved regions, which virtually disappeared over the span of a few microseconds, indicating that clusters of persistent curvature did not develop (Fig. S2). In line with this observation, lipid curvature-partitioning analysis of the unbuckled membrane revealed smaller average curvature values for the different lipids relative to their values in buckled membranes (Figs. S3 and 5 A). While areas of particular curvature appeared and disappeared on the microsecond timescale in the unbuckled system, the mean curvature values of lipids were highly consistent with the trends in a buckled membrane

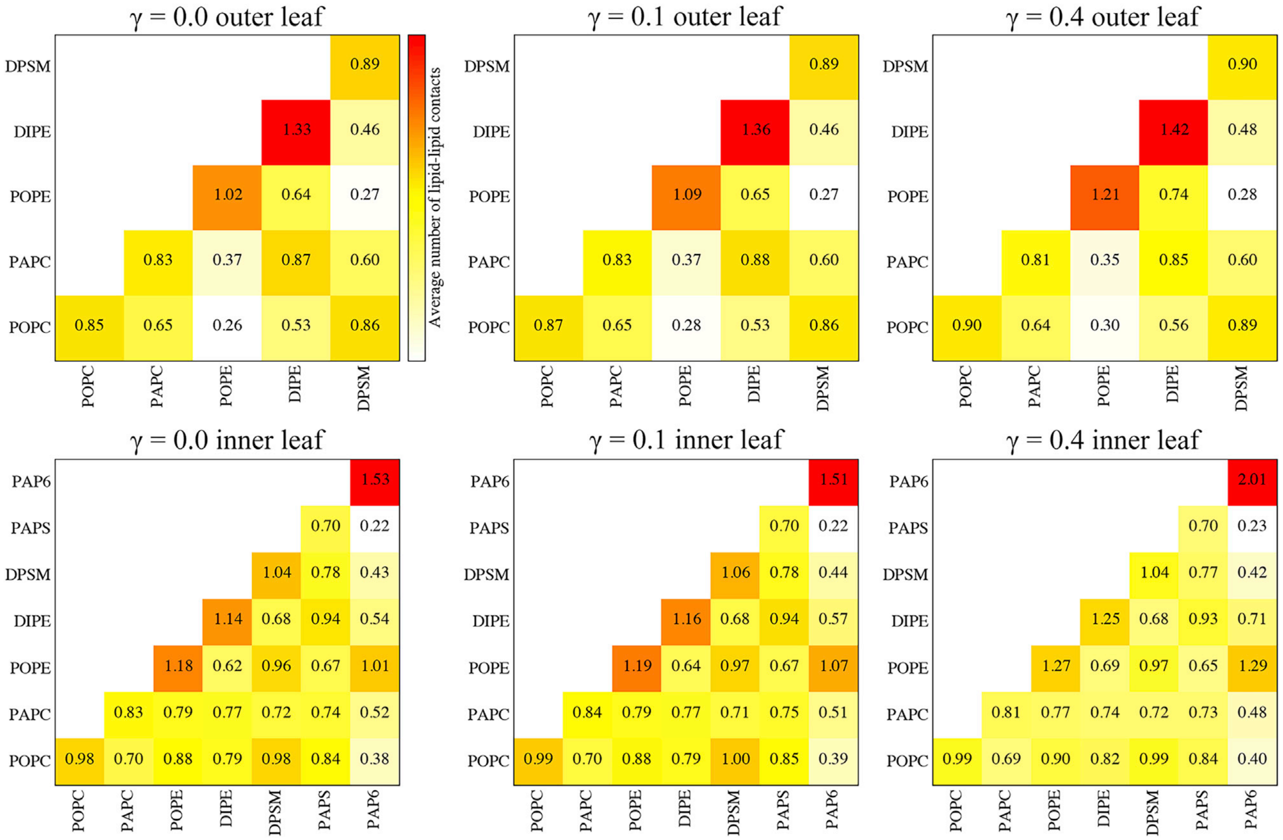

FIGURE 6 Lipid-lipid interactions in unbuckled and buckled membranes. Numbers and shading reflect the average number of contacts between lipids. The cutoff length for counting contacts was 1.0 nm, and the values were normalized by the number and compositional fractions of each pair of lipids (Table 1). Values with their associated uncertainties are given in Tables S1–S6. To see this figure in color, go online.

($r = 0.97$; Fig. S3), though there were some noteworthy differences. In the buckled membrane, PAP6 and POPE exhibited a preference for negatively curved regions (Fig. 5), which remained the case in the unbuckled system, but DIPE and CHOL also showed more distinct accumulation in areas of negative curvature (Fig. S3). A likely explanation is that the unbuckled membrane has a greater area that can assume particular curvatures, whereas the buckled system is more constrained in this sense. Although some of the sorting trends observed in the unbuckled membrane system were consistent with those of spontaneous curvature, such as the difference between PE and PC lipids (64–66), it is important to realize that other factors are at play that can lead to lipids occupying curvature environments that may not coincide with their spontaneous curvatures, for example competition in a heterogeneous lipid mixture.

CONCLUSIONS

Over the years, the fluid mosaic model of cell membranes has advanced to account for observations such as asymmetry and curvature, which are intimately tied to biological function. While curvature-based sorting of proteins and lipids has been reported, and even shown to be directly linked to specific processes in some cases, few studies have considered the molecular details of the phenomenon in membranes with a realistic lipid composition. Simulations at timescales necessary to study curvature-based sorting are currently viable and provide molecular-level details that are crucial for explaining biological processes but that are challenging to obtain experimentally (67,68). This work explored the curvature-based partitioning of eight types of lipids in an asymmetric mammalian bilayer model. Some of the lipids had clear curvature-sorting behavior, while for others it was less evident, though it is crucial to realize that the contributions of factors such as competition for preferred curvature, lipid-lipid interactions, and interleaflet coupling are challenging to discern when interpreting the data. The results are expected to improve our understanding of the role of curvature in membrane organization, and the reported curvature preferences will be useful references for future studies, for instance, in those investigating the functional roles of specific lipids.

SUPPORTING MATERIAL

Supporting material can be found online at https://doi.org/10.1016/j.bpj.2022.05.002.

AUTHOR CONTRIBUTIONS

D.P.T. and E.A.C. designed the research. E.A.C. performed the research and analyzed the data. E.A.C. and D.P.T. wrote the manuscript.

DECLARATION OF INTERESTS

The authors declare no competing interests.

ACKNOWLEDGMENTS

This work was funded by the Natural Sciences and Engineering Research Council of Canada (Canada), grant no. RGPIN/04209-2020. Further support came from the Canada Research Chairs program. Calculations were carried out on Compute Canada machines, funded by the Canada Foundation for Innovation and partners.

REFERENCES

1. Watson, H. 2015. Biological membranes. *Essays Biochem.* 59:43–69. https://doi.org/10.1042/bse0590043.
2. Harayama, T., and H. Riezman. 2018. Understanding the diversity of membrane lipid composition. *Nat. Rev. Mol. Cell Biol.* 19:281–296. https://doi.org/10.1038/nrm.2017.138.
3. Elías-Wolff, F., M. Lindén, …, E. G. Brandt. 2018. Computing curvature sensitivity of biomolecules in membranes by simulated buckling. *J. Chem. Theor. Comput.* 14:1643–1655. https://doi.org/10.1021/acs.jctc.7b00878.
4. Bohuszewicz, O., J. Liu, and H. H. Low. 2016. Membrane remodelling in bacteria. *J. Struct. Biol.* 196:3–14. https://doi.org/10.1016/j.jsb.2016.05.010.
5. Paumard, P., J. Vaillier, …, J. Velours. 2002. The ATP synthase is involved in generating mitochondrial cristae morphology. *EMBO J.* 21:221–230. https://doi.org/10.1093/emboj/21.3.221.
6. Pribil, M., O. Sandoval-Ibáñez, …, D. Leister. 2018. Fine-tuning of photosynthesis requires curvature thylakoid1-mediated thylakoid plasticity. *Plant Physiol.* 176:2351–2364. https://doi.org/10.1104/pp.17.00863.
7. McMahon, H. T., and E. Boucrot. 2015. Membrane curvature at a glance. *J. Cell Sci.* 128:1065–1070. https://doi.org/10.1242/jcs.114454.
8. Prinz, W. A., and J. E. Hinshaw. 2009. Membrane-bending proteins. *Crit. Rev. Biochem. Mol. Biol.* 44:278–291. https://doi.org/10.1080/10409230903183472.
9. Zimmerberg, J., and S. McLaughlin. 2004. Membrane curvature: how BAR domains bend bilayers. *Curr. Biol.* 14:R250–R252. https://doi.org/10.1016/j.cub.2004.02.060.
10. Rilla, K. 2021. Diverse plasma membrane protrusions act as platforms for extracellular vesicle shedding. *J. Extracell. Vesicles.* 10:e12148. https://doi.org/10.1002/jev2.12148.
11. Pike, L. J. 2009. The challenge of lipid rafts. *J. Lipid Res.* 50:S323–S328. https://doi.org/10.1194/jlr.r800040-jlr200.
12. Meinhardt, S., R. L. C. Vink, and F. Schmid. 2013. Monolayer curvature stabilizes nanoscale raft domains in mixed lipid bilayers. *Proc. Natl. Acad. Sci. U. S. A.* 110:4476–4481. https://doi.org/10.1073/pnas.1221075110.
13. Simons, K., and G. Van Meer. 1988. Lipid sorting in epithelial cells. *Biochemistry.* 27:6197–6202. https://doi.org/10.1021/bi00417a001.
14. Roux, A., D. Cuvelier, …, B. Goud. 2005. Role of curvature and phase transition in lipid sorting and fission of membrane tubules. *EMBO J.* 24:1537–1545. https://doi.org/10.1038/sj.emboj.7600631.
15. van Hilten, N., K. S. Stroh, and H. J. Risselada. 2020. Membrane thinning induces sorting of lipids and the amphipathic lipid packing sensor (ALPS) protein motif. *Front. Physiol.* 11:250. https://doi.org/10.3389/fphys.2020.00250.
16. Blum, T. B., A. Hahn, …, W. Kühlbrandt. 2019. Dimers of mitochondrial ATP synthase induce membrane curvature and self-assemble into rows. *Proc. Natl. Acad. Sci. U. S. A.* 116:4250–4255. https://doi.org/10.1073/pnas.1816556116.
17. Kühlbrandt, W. 2015. Structure and function of mitochondrial membrane protein complexes. *BMC Biol.* 13:89. https://doi.org/10.1186/s12915-015-0201-x.
18. Davies, K. M., C. Anselmi, …, W. Kühlbrandt. 2012. Structure of the yeast F1Fo-ATP synthase dimer and its role in shaping the mitochondrial cristae. *Proc. Natl. Acad. Sci. U. S. A.* 109:13602–13607. https://doi.org/10.1073/pnas.1204593109.
19. Nirody, J. A., I. Budin, and P. Rangamani. 2020. ATP synthase: evolution, energetics, and membrane interactions. *J. Gen. Physiol.* 152:e201912475. https://doi.org/10.1085/jgp.201912475.
20. Bozelli, J. C., W. Jennings, …, R. M. Epand. 2018. Membrane curvature allosterically regulates the phosphatidylinositol cycle, controlling its rate and acyl-chain composition of its lipid intermediates. *J. Biol. Chem.* 293:17780–17791. https://doi.org/10.1074/jbc.ra118.005293.
21. Soubias, O., W. E. Teague, …, K. Gawrisch. 2010. Contribution of membrane elastic energy to rhodopsin function. *Biophys. J.* 99:817–824. https://doi.org/10.1016/j.bpj.2010.04.068.
22. Golani, G., N. Ariotti, …, M. M. Kozlov. 2019. Membrane curvature and tension control the formation and collapse of caveolar superstructures. *Dev. Cell.* 48:523–538.e4. https://doi.org/10.1016/j.devcel.2018.12.005.
23. Brown, M. F. 2012. Curvature forces in membrane lipid-protein interactions. *Biochemistry.* 51:9782–9795. https://doi.org/10.1021/bi301332v.
24. Iversen, L., S. Mathiasen, …, D. Stamou. 2015. Membrane curvature bends the laws of physics and chemistry. *Nat. Chem. Biol.* 11:822–825. https://doi.org/10.1038/nchembio.1941.
25. Ingólfsson, H. I., H. Bhatia, …, T. S. Carpenter. 2020. Capturing biologically complex tissue-specific membranes at different levels of compositional complexity. *J. Phys. Chem. B.* 124:7819–7829. https://doi.org/10.1021/acs.jpcb.0c03368.
26. Wassenaar, T. A., H. I. Ingólfsson, …, S. J. Marrink. 2015. Computational lipidomics with insane: a versatile tool for generating custom membranes for molecular simulations. *J. Chem. Theor. Comput.* 11:2144–2155. https://doi.org/10.1021/acs.jctc.5b00209.
27. Ingólfsson, H. I., M. N. Melo, …, S. J. Marrink. 2014. Lipid organization of the plasma membrane. *J. Am. Chem. Soc.* 136:14554–14559. https://doi.org/10.1021/ja507832e.
28. Ingólfsson, H. I., T. S. Carpenter, …, F. C. Lightstone. 2017. Computational lipidomics of the neuronal plasma membrane. *Biophys. J.* 113:2271–2280. https://doi.org/10.1016/j.bpj.2017.10.017.
29. Park, S., A. H. Beaven, …, W. Im. 2015. How tolerant are membrane simulations with mismatch in area per lipid between leaflets? *J. Chem. Theor. Comput.* 11:3466–3477. https://doi.org/10.1021/acs.jctc.5b00232.
30. Hossein, A., and M. Deserno. 2020. Spontaneous curvature, differential stress, and bending modulus of asymmetric lipid membranes. *Biophys. J.* 118:91a–642. https://doi.org/10.1016/j.bpj.2019.11.661.
31. De Jong, D. H., G. Singh, …, S. J. Marrink. 2013. Improved parameters for the martini coarse-grained protein force field. *J. Chem. Theor. Comput.* 9:687–697. https://doi.org/10.1021/ct300646g.
32. Wang, Y., P. Gkeka, …, Z. Cournia. 2016. DPPC-cholesterol phase diagram using coarse-grained molecular dynamics simulations. *Biochim. Biophys. Acta - Biomembr.* 1858:2846–2857. https://doi.org/10.1016/j.bbamem.2016.08.005.
33. Abraham, M. J., T. Murtola, …, E. Lindahl. 2015. GROMACS: high performance molecular simulations through multi-level parallelism from laptops to supercomputers. *SoftwareX.* 1–2:19–25. https://doi.org/10.1016/j.softx.2015.06.001.

34. De Jong, D. H., S. Baoukina, …, S. J. Marrink. 2016. Martini straight: boosting performance using a shorter cutoff and GPUs. *Comput. Phys. Commun.* 199:1–7. https://doi.org/10.1016/j.cpc.2015.09.014.

35. Bussi, G., D. Donadio, and M. Parrinello. 2007. Canonical sampling through velocity rescaling. *J. Chem. Phys.* 126:014101. https://doi.org/10.1063/1.2408420.

36. Parrinello, M., and A. Rahman. 1981. Polymorphic transitions in single crystals: a new molecular dynamics method. *J. Appl. Phys.* 52:7182–7190. https://doi.org/10.1063/1.328693.

37. Cino, E. A., M. Borbuliak, …, D. P. Tieleman. 2021. Lipid distributions and transleaflet cholesterol migration near heterogeneous surfaces in asymmetric bilayers. *Faraday Discuss.* 232:103–113. https://doi.org/10.1039/d1fd00003a.

38. Doktorova, M., D. Harries, and G. Khelashvili. 2017. Determination of bending rigidity and tilt modulus of lipid membranes from real-space fluctuation analysis of molecular dynamics simulations. *Phys. Chem. Chem. Phys.* 19:16806–16818. https://doi.org/10.1039/c7cp01921a.

39. Johner, N., D. Harries, and G. Khelashvili. 2016. Implementation of a methodology for determining elastic properties of lipid assemblies from molecular dynamics simulations. *BMC Bioinformatics.* 17, 161. https://doi.org/10.1186/s12859-016-1003-z.

40. Gapsys, V., B. L. De Groot, and R. Briones. 2013. Computational analysis of local membrane properties. *J. Comput. Aided. Mol. Des.* 27:845–858. https://doi.org/10.1007/s10822-013-9684-0.

41. Allen, W. J., J. A. Lemkul, and D. R. Bevan. 2009. GridMAT-MD: a grid-based membrane analysis tool for use with molecular dynamics. *J. Comput. Chem.* 30:1952–1958. https://doi.org/10.1002/jcc.21172.

42. Boyd, K. J., N. N. Alder, and E. R. May. 2017. Buckling under pressure: curvature-based lipid segregation and stability modulation in cardiolipin-containing bilayers. *Langmuir.* 33:6937–6946. https://doi.org/10.1021/acs.langmuir.7b01185.

43. Mironov, A. A., A. Mironov, G. V. Beznoussenko…, 2020. Membrane curvature, trans-membrane area asymmetry, budding, fission and organelle geometry. *Int. J. Mol. Sci.* 21, 7594. https://doi.org/10.3390/ijms21207594.

44. Scott, H. L., K. B. Kennison, …, J. Katsaras. 2021. Model membrane systems used to study plasma membrane lipid asymmetry. *Symmetry (Basel).* 13:1356. https://doi.org/10.3390/sym13081356.

45. Dasgupta, R., M. S. Miettinen, …, R. Dimova. 2018. The glycolipid GM1 reshapes asymmetric biomembranes and giant vesicles by curvature generation. *Proc. Natl. Acad. Sci. U. S. A.* 115:5756–5761. https://doi.org/10.1073/pnas.1722320115.

46. Elani, Y., S. Purushothaman, …, O. Ces. 2015. Measurements of the effect of membrane asymmetry on the mechanical properties of lipid bilayers. *Chem. Commun.* 51:6976–6979. https://doi.org/10.1039/c5cc00712g.

47. Lu, L., W. J. Doak, …, P. R. Chiarot. 2016. Membrane mechanical properties of synthetic asymmetric phospholipid vesicles. *Soft Matter.* 12:7521–7528. https://doi.org/10.1039/c6sm01349j.

48. Vorselen, D., F. C. Mackintosh, …, G. J. Wuite. 2017. Competition between bending and internal pressure governs the mechanics of fluid nanovesicles. *ACS Nano.* 11:2628–2636. https://doi.org/10.1021/acsnano.6b07302.

49. Kondrashov, O. V., T. R. Galimzyanov, S. A. Akimov…, 2020. Membrane-mediated lateral interactions regulate the lifetime of gramicidin channels. *Membranes (Basel).* 10, 368. https://doi.org/10.3390/membranes10120368.

50. Park, Y. K., C. A. Best, …, G. Popescu. 2010. Measurement of red blood cell mechanics during morphological changes. *Proc. Natl. Acad. Sci. U. S. A.* 107:6731–6736. https://doi.org/10.1073/pnas.0909533107.

51. Beltrán-Heredia, E., F. C. Tsai, …, F. Monroy. 2019. Membrane curvature induces cardiolipin sorting. *Commun. Biol.* 2:1–7.

52. Koldsø, H., D. Shorthouse, …, M. S. P. Sansom. 2014. Lipid clustering correlates with membrane curvature as revealed by molecular simulations of complex lipid bilayers. *PLoS Comput. Biol.* 10:1003911. https://doi.org/10.1371/journal.pcbi.1003911.

53. Elías-Wolff, F., M. LindénSweden, …, E. G. Brandt. 2019. Curvature sensing by cardiolipin in simulated buckled membranes. *Soft Matter.* 15:792–802. https://doi.org/10.1039/c8sm02133c.

54. Chen, Z., and R. P. Rand. 1997. The influence of cholesterol on phospholipid membrane curvature and bending elasticity. *Biophys. J.* 73:267–276. https://doi.org/10.1016/s0006-3495(97)78067-6.

55. Baoukina, S., H. I. Ingólfsson, …, D. P. Tieleman. 2018. Curvature-induced sorting of lipids in plasma membrane tethers. *Adv. Theor. Simulations.* 1:1800034. https://doi.org/10.1002/adts.201800034.

56. Daily, M. D., B. N. Olsen, …, N. A. Baker. 2014. Improved coarse-grained modeling of cholesterol-containing lipid bilayers. *J. Chem. Theor. Comput.* 10:2137–2150. https://doi.org/10.1021/ct401028g.

57. Callan-Jones, A., B. Sorre, and P. Bassereau. 2011. Curvature-driven lipid sorting in biomembranes. *Cold Spring Harb. Perspect. Biol.* 3:1–14.

58. Bennett, W. D., and D. P. Tieleman. 2013. Computer simulations of lipid membrane domains. *Biochim. Biophys. Acta - Biomembr.* 1828:1765–1776. https://doi.org/10.1016/j.bbamem.2013.03.004.

59. Wang, J., and D. A. Richards. 2012. Segregation of PIP2 and PIP3 into distinct nanoscale regions within the plasma membrane. *Biol. Open.* 1:857–862. https://doi.org/10.1242/bio.20122071.

60. Van Den Bogaart, G., K. Meyenberg, …, R. Jahn. 2011. Membrane protein sequestering by ionic protein-lipid interactions. *Nature.* 479:552–555. https://doi.org/10.1038/nature10545.

61. Kimble-Hill, A. C., H. I. Petrache, …, M. A. Firestone. 2018. Reorganization of ternary lipid mixtures of nonphosphorylated phosphatidylinositol interacting with angiomotin. *J. Phys. Chem. B.* 122:8404–8415. https://doi.org/10.1021/acs.jpcb.7b12641.

62. Sarmento, M. J., M. Hof, and R. Šachl. 2020. Interleaflet coupling of lipid nanodomains – insights from *in vitro* systems. *Front. Cell Dev. Biol.* 8:284. https://doi.org/10.3389/fcell.2020.00284.

63. Eicher, B., D. Marquardt, …, G. Pabst. 2018. Intrinsic curvature-mediated transbilayer coupling in asymmetric lipid vesicles. *Biophys. J.* 114:146–157. https://doi.org/10.1016/j.bpj.2017.11.009.

64. Allender, D. W., H. Giang, and M. Schick. 2020. Model plasma membrane exhibits a microemulsion in both leaves providing a foundation for "rafts. *Biophys. J.* 118:1019–1031. https://doi.org/10.1016/j.bpj.2020.01.004.

65. Sodt, A. J., R. Pastor, and R. W. Pastor. 2013. Bending free energy from simulation: correspondence of planar and inverse hexagonal lipid phases. *Biophys. J.* 104:2202–2211. https://doi.org/10.1016/j.bpj.2013.03.048.

66. Sapp, K. C., A. H. Beaven, and A. J. Sodt. 2021. Spatial extent of a single lipid's influence on bilayer mechanics. *Phys. Rev. E.* 103:042413. https://doi.org/10.1103/physreve.103.042413.

67. Corradi, V., E. Mendez-Villuendas, …, D. P. Tieleman. 2018. Lipid-protein interactions are unique fingerprints for membrane proteins. *ACS Cent. Sci.* 4:709–717. https://doi.org/10.1021/acscentsci.8b00143.

68. Tieleman, D. P., B. I. Sejdiu, …, V. Corradi. 2021. Insights into lipid-protein interactions from computer simulations. *Biophys. Rev.* 13:1019–1027. https://doi.org/10.1007/s12551-021-00876-9.

Biophysical Journal, Volume *121*

Supplemental information

Curvature-based sorting of eight lipid types in asymmetric buckled plasma membrane models

Elio A. Cino and D. Peter Tieleman

Curvature-based sorting of eight lipid types in asymmetric buckled plasma membrane models

Elio A. Cino[1], D. Peter Tieleman[1*]

[1]Centre for Molecular Simulation and Department of Biological Sciences, University of Calgary, 2500 University Drive NW, Calgary, Alberta T2N 1N4, Canada

***D. Peter Tieleman, tieleman@ucalgary.ca**

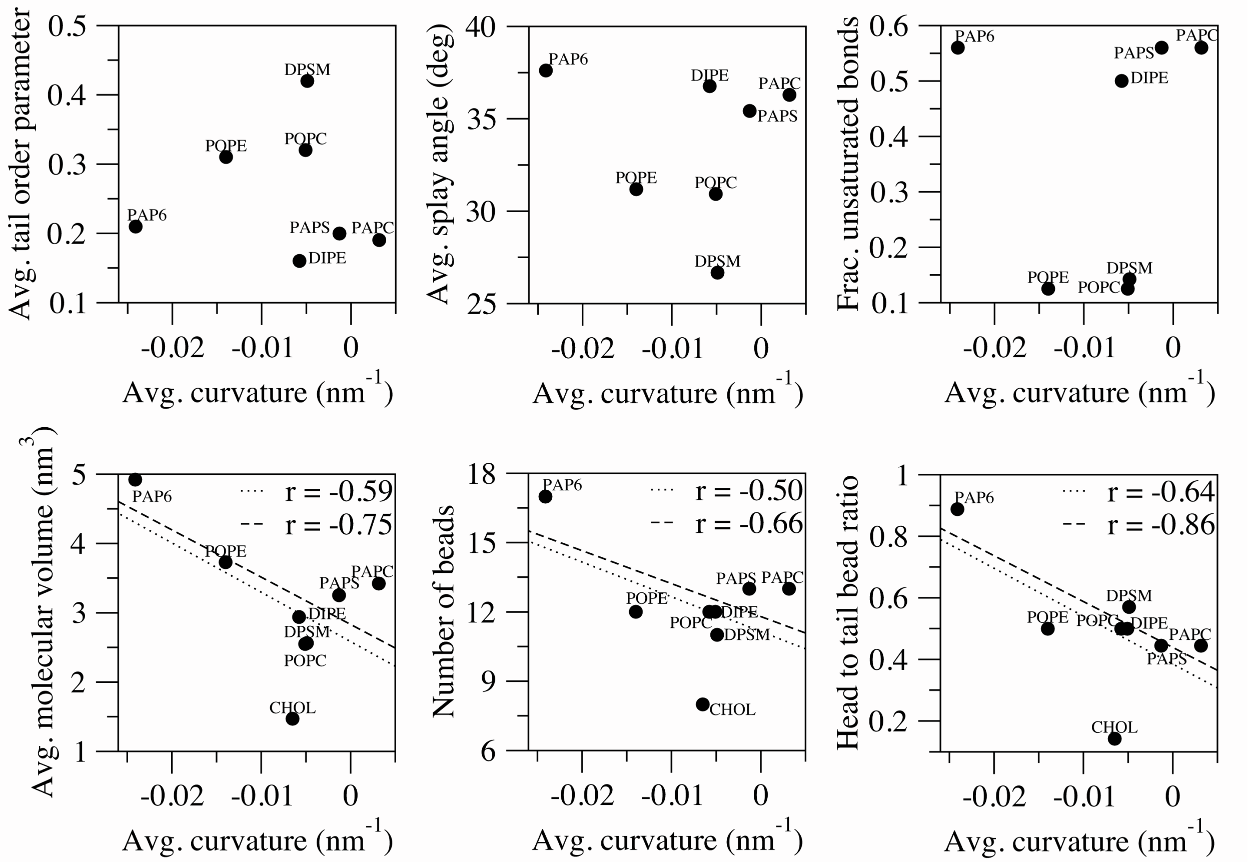

Figure S1. Correlations between average curvatures and various parameters. Splay angles were defined by the terminal beads of each tail and PO4 of the headgroup, similar to (1). Absolute lipid tail order parameters (average of the tail beads) were calculated using the do-order-gmx5.py script from the Martini website. Approximate lipid molecular volumes were computed using gmx sasa with a probe radius of 0.26 nm, which corresponds to the bead vdW radius (2). The values were systematically higher than reported from experiment (3); however the relative values are consistent with the molecular structures (Fig. 1), and similar trends were obtained using the number of beads instead of volumes. Dotted and dashed lines correspond to fits including and excluding CHOL, respectively.

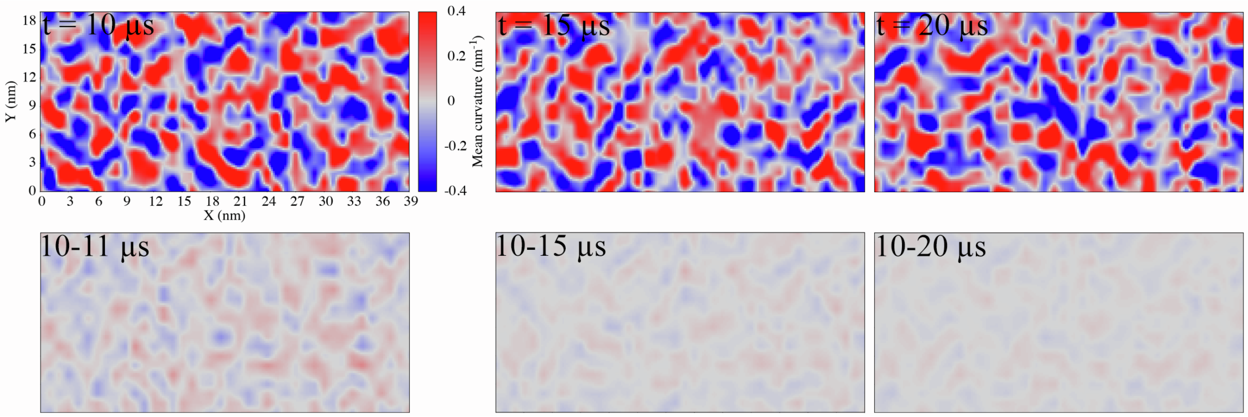

Figure S2. Mean curvature maps of the outer leaf of the unbuckled system. Single frame calculations at 10, 15 and 20 μs (top), and averaging over 1, 5, and 10 μs time intervals (bottom).

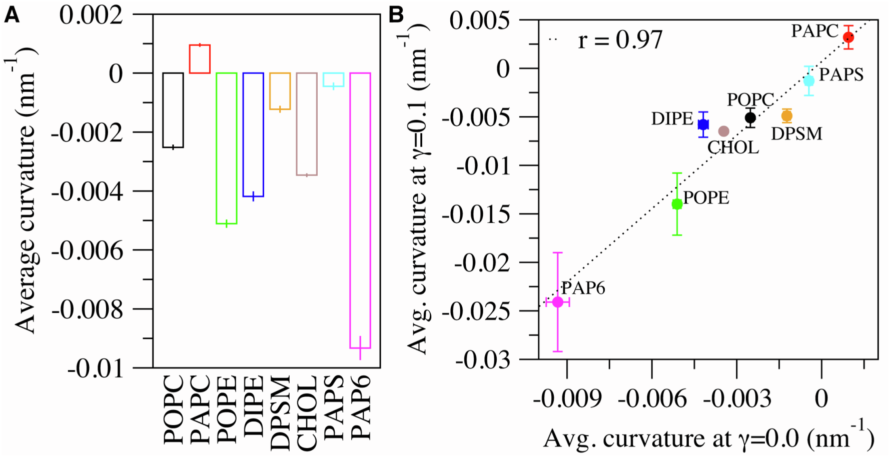

Figure S3. Curvature values of lipids in the unbuckled system and comparison with a buckled system. A) Mean curvature values of lipids in the unbuckled, γ=0.0 system. B) Comparison of the curvature values from the γ=0.0 and γ=0.1 systems.

Table S1: Average number of lipid-lipid contacts in the γ=0.0 outer leaf.

	POPC	PAPC	POPE	DIPE	DPSM
POPC	0.85±0.01	0.65±0.01	0.26±0.01	0.53±0.01	0.86±0.01
PAPC		0.83±0.01	0.37±0.02	0.87±0.02	0.60±0.01
POPE			1.02±0.03	0.64±0.02	0.27±0.01
DIPE				1.33±0.04	0.46±0.01
DPSM					0.89±0.01

Table S2: Average number of lipid-lipid contacts in the γ=0.0 inner leaf.

	POPC	PAPC	POPE	DIPE	DPSM	PAPS	PAP6
POPC	0.98±0.01	0.70±0.01	0.88±0.01	0.79±0.01	0.98±0.01	0.84±0.01	0.38±0.01
PAPC		0.83±0.01	0.79±0.02	0.77±0.01	0.72±0.01	0.74±0.01	0.52±0.02
POPE			1.18±0.03	0.62±0.01	0.96±0.02	0.67±0.01	1.01±0.05
DIPE				1.14±0.01	0.68±0.01	0.94±0.01	0.54±0.01
DPSM					1.04±0.02	0.78±0.01	0.43±0.02
PAPS						0.70±0.01	0.22±0.01
PAP6							1.53±0.09

Table S3: Average number of lipid-lipid contacts in the γ=0.1 outer leaf.

	POPC	PAPC	POPE	DIPE	DPSM
POPC	0.87±0.01	0.65±0.01	0.28±0.01	0.53±0.01	0.86±0.01
PAPC		0.83±0.02	0.37±0.02	0.88±0.02	0.60±0.01
POPE			1.09±0.24	0.65±0.03	0.27±0.01
DIPE				1.36±0.08	0.46±0.01
DPSM					0.89±0.01

Table S4: Average number of lipid-lipid contacts in the γ=0.1 inner leaf.

	POPC	PAPC	POPE	DIPE	DPSM	PAPS	PAP6
POPC	0.99±0.01	0.70±0.01	0.88±0.01	0.79±0.01	1.00±0.01	0.85±0.01	0.39±0.02
PAPC		0.84±0.02	0.79±0.01	0.77±0.01	0.71±0.01	0.75±0.01	0.51±0.01
POPE			1.19±0.03	0.64±0.01	0.97±0.03	0.67±0.01	1.07±0.06
DIPE				1.16±0.01	0.68±0.01	0.94±0.01	0.57±0.03
DPSM					1.06±0.01	0.78±0.01	0.44±0.03
PAPS						0.70±0.01	0.22±0.01
PAP6							1.51±0.14

Table S5: Average number of lipid-lipid contacts in the γ=0.4 outer leaf.

	POPC	PAPC	POPE	DIPE	DPSM
POPC	0.90±0.01	0.64±0.01	0.30±0.01	0.56±0.01	0.89±0.01
PAPC		0.81±0.02	0.35±0.01	0.85±0.02	0.60±0.01
POPE			1.21±0.18	0.74±0.05	0.28±0.01
DIPE				1.42±0.04	0.48±0.01
DPSM					0.90±0.01

Table S6: Average number of lipid-lipid contacts in the γ=0.4 inner leaf.

	POPC	PAPC	POPE	DIPE	DPSM	PAPS	PAP6
POPC	0.99±0.01	0.69±0.01	0.90±0.01	0.82±0.01	0.99±0.01	0.84±0.01	0.40±0.01
PAPC		0.81±0.01	0.77±0.02	0.74±0.01	0.72±0.01	0.73±0.01	0.48±0.03
POPE			1.27±0.08	0.69±0.02	0.97±0.01	0.65±0.01	1.29±0.17
DIPE				1.25±0.04	0.68±0.01	0.93±0.01	0.71±0.04
DPSM					1.04±0.02	0.77±0.01	0.42±0.03
PAPS						0.70±0.01	0.23±0.01
PAP6							2.01±0.32

References

1. Kawamoto, S., M.L. Klein, and W. Shinoda. 2015. Coarse-grained molecular dynamics study of membrane fusion: Curvature effects on free energy barriers along the stalk mechanism. *J. Chem. Phys.* 143:243112.
2. Periole, X., A.M. Knepp, T.P. Sakmar, S.J. Marrink, and T. Huber. 2012. Structural determinants of the supramolecular organization of G protein-coupled receptors in bilayers. *J. Am. Chem. Soc.* 134:10959–10965.
3. Marsh, D. 2010. Molecular volumes of phospholipids and glycolipids in membranes. *Chem. Phys. Lipids*. 163:667–677.
